# Peri-Intubation Cardiac Arrest in a Patient With Hypertensive Cardiac Tamponade

**DOI:** 10.7759/cureus.76178

**Published:** 2024-12-22

**Authors:** Jamiu O Aliu, Marta Montero, Jessica Dorman

**Affiliations:** 1 Intensive Care Unit, Salford Royal NHS Foundation Trust, Salford, GBR; 2 Intensive Care Unit, Newham University Hospital, Barts Health NHS Trust, London, GBR; 3 Emergency Medicine, Barts Health NHS Trust, London, GBR

**Keywords:** cardiac arrest, cardiac tamponade, intubation, pericardial effusion, pericardiocentesis

## Abstract

Cardiac tamponade is widely known to be associated with life-threatening hypotension and rarely with hypertension. We present the case of a 53-year-old woman with hypertension and echocardiographic features of tamponade who had a cardiac arrest on intubation. The paradoxical hypertension is thought to be due to tachycardia and increased peripheral vascular resistance resulting from the compensatory sympathetic surge following impaired cardiac filling caused by the effusion. In the event of cardiorespiratory failure, pericardiocentesis should be considered before intubation in this set of patients.

## Introduction

Cardiac tamponade is widely known to be associated with symptoms such as pleuritic chest pain, palpitation, shortness of breath, and obstructive shock, which can be life-threatening [1]. Although making the diagnosis of tamponade with clinical signs alone is difficult since they are neither sensitive nor specific [2], hypotension is regarded as a hallmark feature, and despite there being reports of its possibility [3,4], the co-existence of cardiac tamponade and high blood pressure (BP) is rarely discussed in clinical practice. We present the case of a 53-year-old woman who was a clear deviation from the norm; she had acute respiratory failure and hypertension with echocardiographic features of cardiac tamponade and went into cardiac arrest on endotracheal intubation.

## Case presentation

A peri-arrest call was made at about 04:30 for a 53-year-old woman with no known previous medical history who had been admitted to the medical ward for the evaluation of suspected breast cancer. She was known to have mild pleural and moderate pericardial effusion. On initial assessment, she was in obvious respiratory distress, with an oxygen saturation (SpO2) of 85% on a 10 L non-rebreather mask, a respiratory rate (RR) of 50 cycles per minute (CPM), a BP of 184/110 mmHg, and a heart rate (HR) between 130 and 140 beats per minute (BPM). Arterial blood gas (ABG) showed type 1 respiratory failure (T1RF) with a partial pressure of oxygen (pO2) of 7.2 on 15 liters of oxygen via a non-rebreather mask. The electrocardiogram at that time was normal.

**Figure 1:**
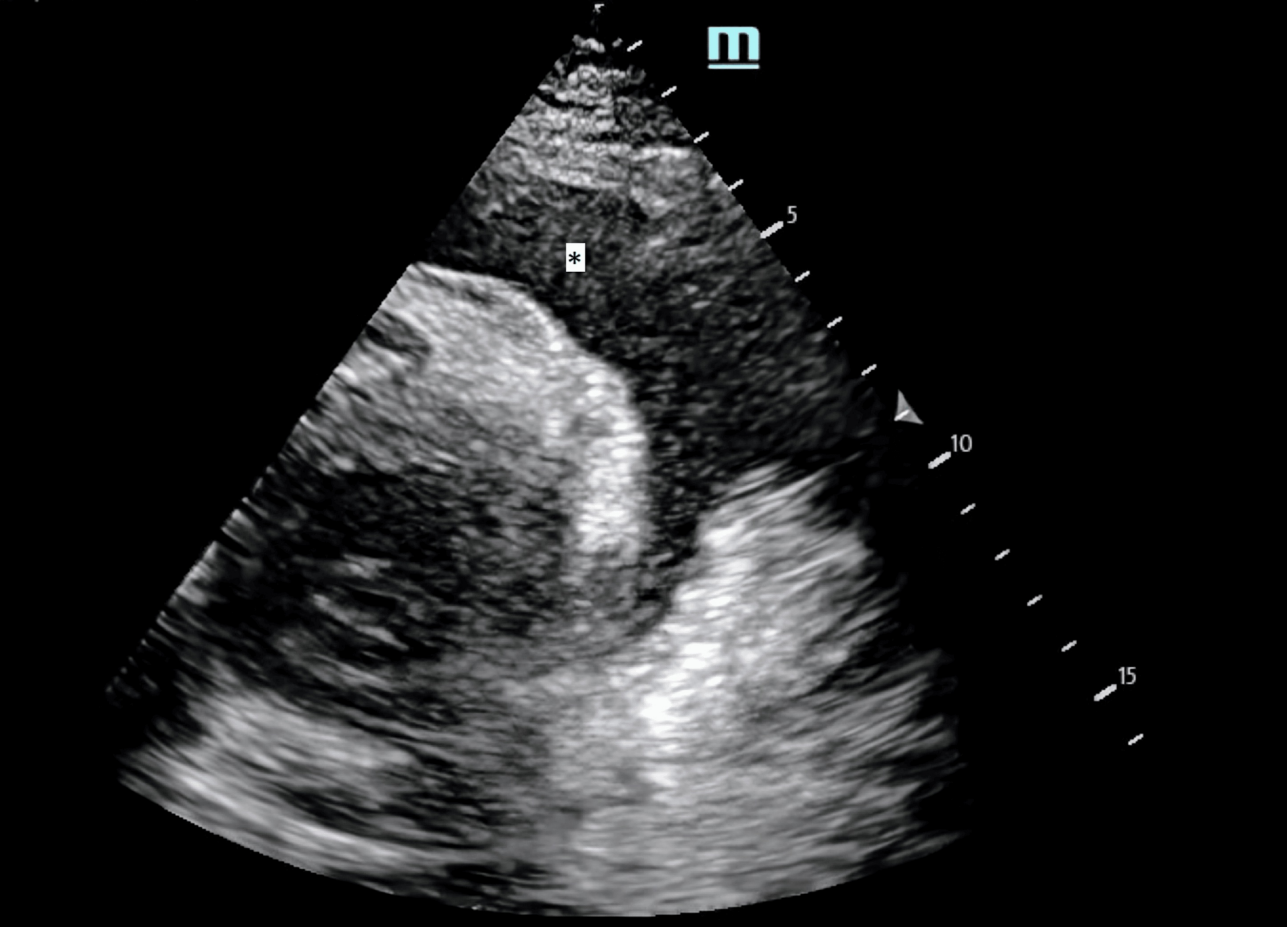
POCUS echocardiogram: parasternal short-axis view 22 mm pericardial effusion (*) around the ventricles. POCUS: point-of-care ultrasound

**Figure 2:**
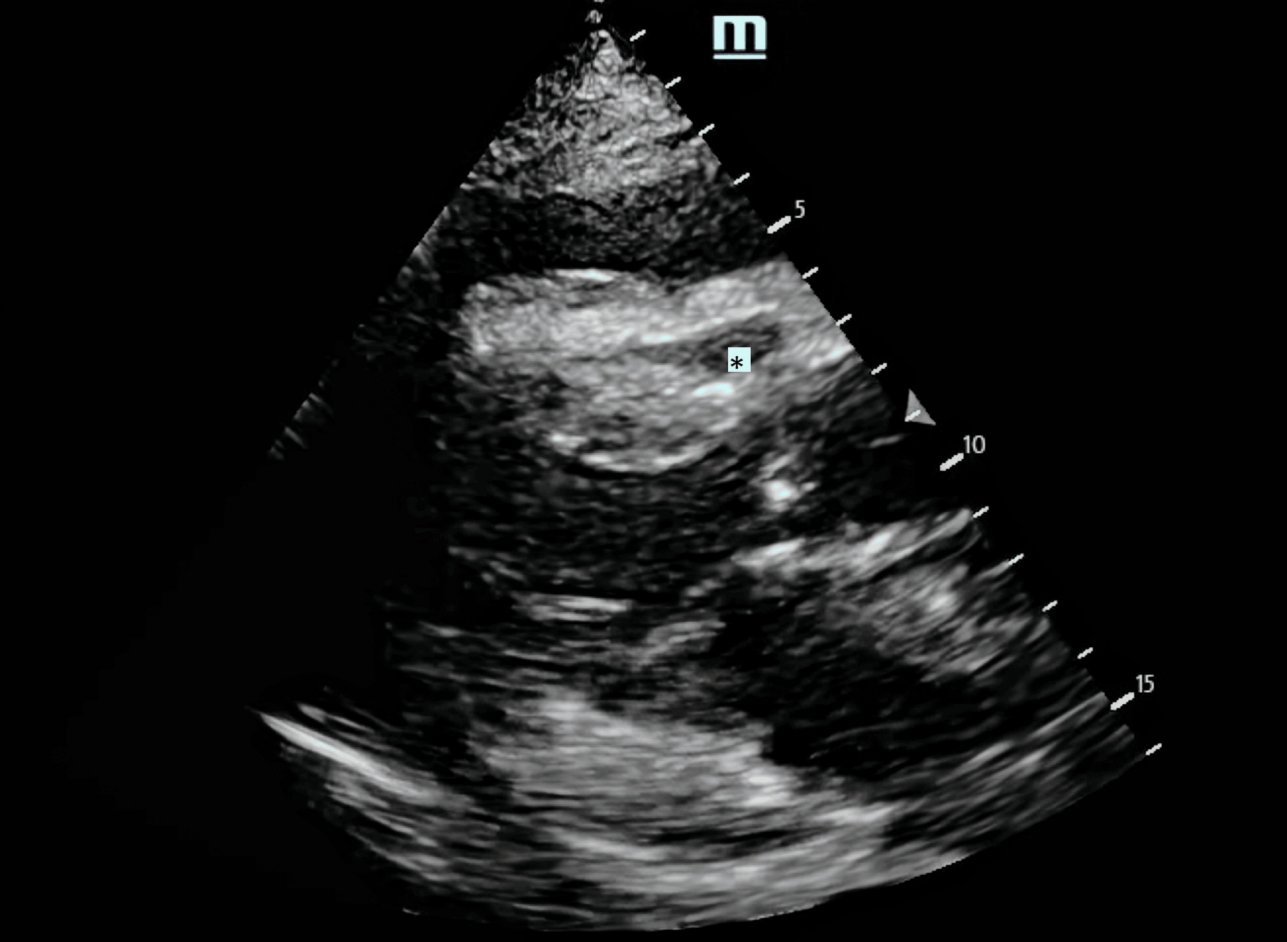
POCUS echocardiogram: parasternal long-axis view Right ventricular diastolic collapse (*), a highly specific sign of cardiac tamponade. POCUS: point-of-care ultrasound

**Figure 3:**
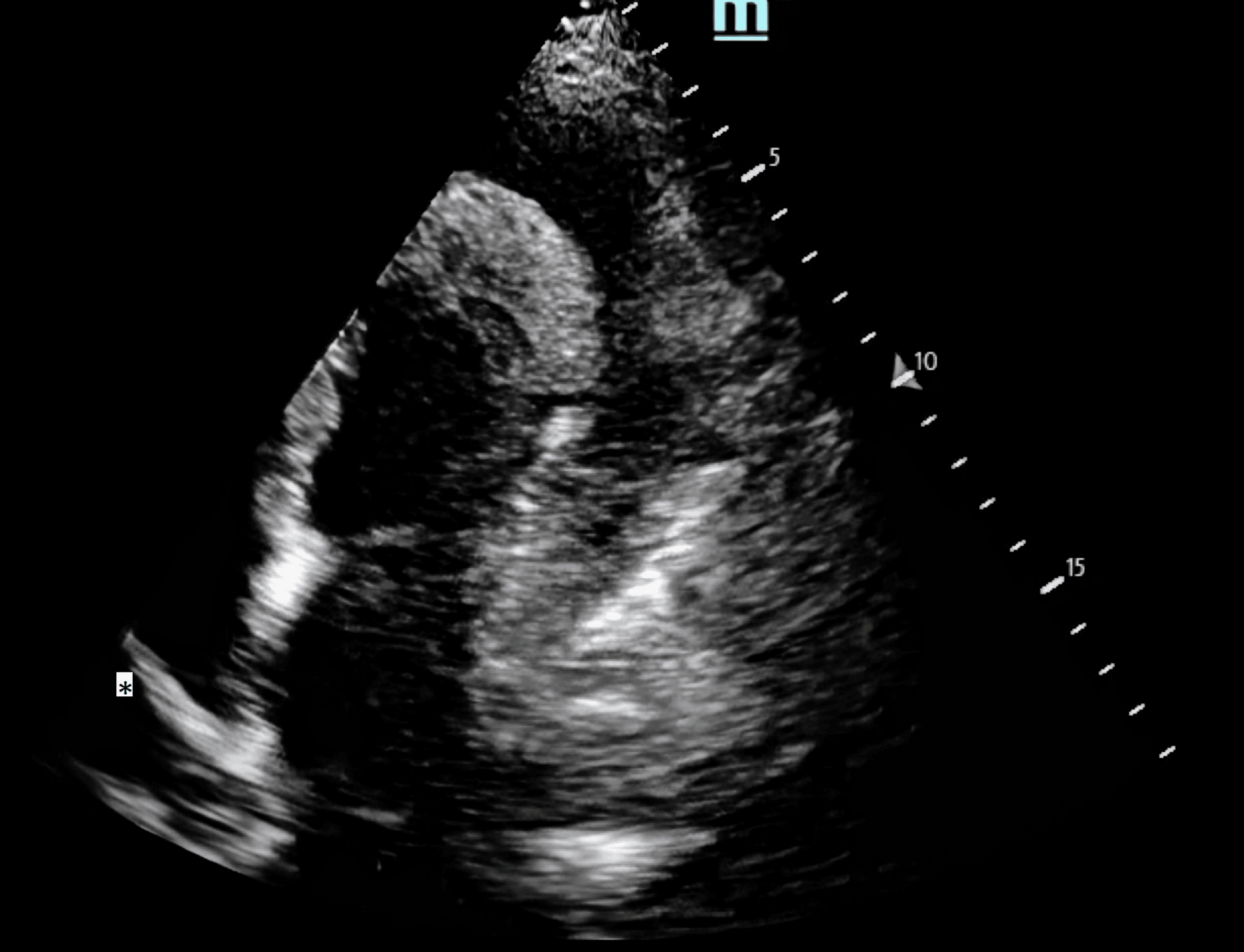
POCUS echocardiogram: apical four-chamber view Right atrial inversion (*), a sign of cardiac tamponade. POCUS: point-of-care ultrasound

**Figure 4:**
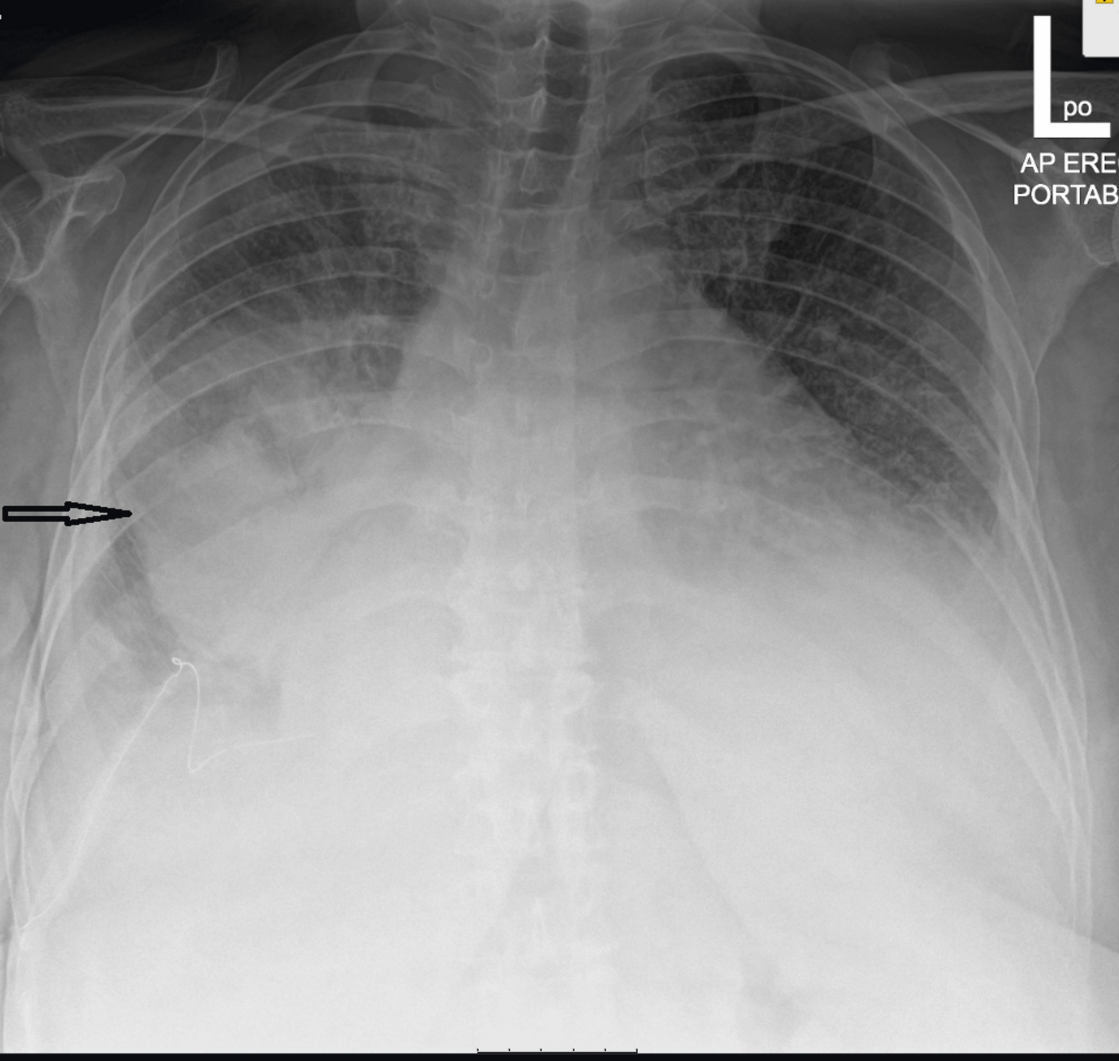
Chest radiograph: antero-posterior view Round opacification of the right mid zone (arrowed). Also, bilateral congestion and pleural effusions and increased cardiothoracic ratio.

The patient had muffled heart sounds and distended neck veins. Point-of-care ultrasound (POCUS) of the heart revealed circumferential pericardial effusion of about 22 mm with echocardiographic features of tamponade (Figures 1-3). This was the same amount described on the formal echocardiogram she had had a week prior. Chest radiograph revealed a dense opacity in the right mid zone and features of pulmonary edema and pleural effusion bilaterally with the lower end of the trachea deviated to the right (Figure 4). She had 80 mg of furosemide STAT but was too agitated to tolerate non-invasive ventilation. The T1RF worsened despite high-flow oxygen therapy. As there had been no significant change in the size of the effusion compared to previous radiographs, and systolic blood pressure (SBP) via invasive arterial BP (ABP) monitoring was consistently above 180, her respiratory system was deemed the most likely cause of the acute deterioration, and endotracheal intubation was thought to be the most appropriate next line of management rather than emergency pericardiocentesis.

Attempts were made at optimization before rapid sequence induction (RSI) as the patient had a high risk of peri-induction instability. An arterial and a central venous line were inserted, and an infusion of Hartmann's solution was started to increase the preload. An airway-trained ICU registrar was the intubator, and an anesthetic registrar was in attendance as well. RSI was done with a standardized checklist; medications used for RSI were fentanyl, midazolam, and rocuronium; and laryngoscopy was performed with a MAC 4-blade on the C-MAC video laryngoscope with first-pass success. BP dropped rapidly seconds after passing the endotracheal tube, and the patient went into cardiac arrest. Return of spontaneous circulation (ROSC) was achieved after three cycles of cardiopulmonary resuscitation, with the patient requiring high-dose noradrenaline infusion to maintain ROSC. Noradrenaline requirement improved, and she was weaned off after 24 hours; she was extubated on supplemental oxygen. After multi-disciplinary team meetings, she was deemed unfit for active treatment and was stepped down with a ward-level ceiling of care.

## Discussion

Hypotension and distended neck veins and muffled heart sounds (Beck's triad) are considered the classical signs of cardiac tamponade [5]. Although uncommon, hypertension can co-exist with tamponade, and the incidence of this, as described in some studies, could be as high as 1-in-3 [3,4]. This phenomenon is usually associated with chronic hypertension and advanced renal disease [4], none of which was present in our patient. There has been no report of it being associated with tamponades due to malignant effusions.

The paradoxical hypertension in cardiac tamponade is thought to be due to tachycardia and increased peripheral vascular resistance (PVR). These two factors result from the compensatory sympathetic surge following impaired cardiac filling caused by the pericardial effusion [6]. This explains the significant decrease in BP after pericardiocentesis in these patients [3,4], as the resultant decreased intracardiac pressure will reduce the sympathetic tone for the patient presented; the possible explanation for the shock is akin to the above. Vasodilation from the RSI medications, reduced venous return from positive end-expiratory pressure, and cardiac tamponade all happening abruptly could lead, as in this case, to a catastrophe.

## Conclusions

Although it is a rare phenomenon, hypertension can co-exist with cardiac tamponade. There have been reports of and explanations for this in the literature, but it is regarded as a niche topic in clinical practice. Beck's triad should not be used in isolation to rule out tamponade, especially with positive echocardiographic features, as it is, alongside other signs, neither sensitive nor specific. Furthermore, emergency pericardiocentesis should be considered before intubation in patients such as the one we have presented. However, we appreciate how much of a dilemma this case presents and how essential it was to address the respiratory failure as quickly as possible.
